# Effect of screening mammography on breast cancer mortality: Quasi‐experimental evidence from rollout of the Dutch population‐based program with 17‐year follow‐up of a cohort

**DOI:** 10.1002/ijc.32584

**Published:** 2019-08-07

**Authors:** Tom Van Ourti, Owen O'Donnell, Hale Koç, Jacques Fracheboud, Harry J. de Koning

**Affiliations:** ^1^ Erasmus School of Economics, Tinbergen Institute Erasmus University Rotterdam Rotterdam The Netherlands; ^2^ Tinbergen Institute Erasmus University Rotterdam Rotterdam The Netherlands; ^3^ Department of Public Health Erasmus MC ‐ University Medical Center Rotterdam Rotterdam The Netherlands

**Keywords:** mammography screening, breast cancer mortality, quasi‐experimental exposure, population administrative data

## Abstract

There is uncertainty about the magnitude of the effect of screening mammography on breast cancer mortality. The relevance and validity of evidence from dated randomized controlled trials has been questioned, whereas observational studies often lack a valid comparison group. There is no estimate of the effect of one screening invitation only. We exploited the geographic rollout of the Dutch screening mammography program across municipalities to estimate the effects of one additional biennial screening invitation on breast cancer and all‐cause mortality. Population administrative data provided vital status and cause of death of a cohort of women aged 49–63 in 1995 over 17 years. Linear probability models were used to estimate the mortality effects. We estimated 154 fewer breast cancer deaths (95% confidence interval: 40–267; *p* = 0.01) over 17 years in a population of 100,000 women aged 49–63 who received one additional biennial screening invitation, which corresponds to an 9.6% risk reduction for a woman of age 56. The estimated effect on all‐cause mortality was negative but not close to statistical significance. Our study shows that one single invitation for breast cancer screening is effective in reducing breast cancer mortality, which is important for health policy. The effect is smaller than previous estimates of the effect of invitation for multiple screens, which further emphasizes the importance of achieving regular participation.

## Abstract 2

**What's new?**

To date, there is still uncertainty about the magnitude of the effect of screening mammography on breast cancer mortality. Here, the authors exploited the geographic rollout of the Dutch screening mammography program and high‐quality national population, cancer, and death registries to avoid limitations of observational research by comparing breast cancer mortality across groups of women of the same age who joined the mammography program at different dates. The analysis provides a unique estimate of the effect of one additional invitation for screening mammography on breast cancer mortality (around 10%) and delivers evidence in favour of the effectiveness of such screening.

## Introduction

It is 35 years since randomized controlled trials (RCTs) appeared to show that mammography screening reduced breast cancer mortality by 25%,^1^, ^2^ and yet there remains debate about the presently achievable magnitude of effect.^3^, ^4^, ^5^, ^6^, ^7^, ^8^, ^9^ Weaknesses in the methodology of several RCTs,^4^ their limited relevance today given advances in treatment, and their inability to establish effectiveness of population‐based screening programs have led to greater reliance on observational studies.^5^ The validity of evidence from such studies^10^, ^11^, ^12^, ^13^, ^14^, ^15^, ^16^ may also be doubted when a comparison group is lacking or inappropriate, the follow‐up period is limited, or potential confounders are not adequately controlled. Comparison of national trends before and after the introduction of organized screening is certainly biased by changes in background risk, increased awareness of breast cancer, and improved effectiveness of treatment.^12^ Comparison of before‐and‐after trends in a group that gains access to screening with trends observed over a different period in a group not covered by screening^13^, ^14^ does not eliminate the effect of these confounders.

Exploiting Dutch population and cancer registries with 100% coverage rate and detailed data on the date the screening mammography program started in each municipality, our study avoided these limitations by comparing breast cancer mortality across two groups of women of the same age observed over the same 17‐year period who got access to the program at different dates due to its staggered rollout. The group that entered the program earlier had received one more invitation for screening in every year of our follow‐up period. Provided breast cancer mortality would not otherwise have differed systematically across the two groups of municipalities, any observed difference in mortality can be attributed to the additional biennial screening invitation. Contrary to most evaluations of screening mammography, we use a cohort analysis which is better suited to distinguishing a screening invitation effect from confounding factors than designs that do not follow the same cohort over time. To our knowledge, this is the first article to identify the effect of one (additional) screen invitation.

## Methods

### Screening program

In 1989, the Netherlands initiated a population‐based, organized screening program for women aged 50 to 69. This was rolled out by municipality following no particular pattern over 9 years. By 1997, almost 800,000 women were targeted and 80% accepted an invitation for screening.^17^ In 1998, the upper age limit was extended to 75. Within 3 years, this extension was implemented nationwide.^18^

All eligible women in a zipcode area simultaneously receive a biennial personalized invitation to get screened in a (mostly) mobile unit at a specific date and time. There is no charge. Mammograms are independently assessed by two radiologists, and a woman is recalled if both agree on a positive result. Clinical assessment and breast cancer treatment is universally available without charge.

### Data and study population

We used municipality and death registers accessed via Statistics Netherlands to identify date of birth, vital status, date and cause of death, and zipcode for every female resident of the Netherlands from January 1995 to December 2011. From the Dutch Cancer Registry, we retrieved municipality‐level breast cancer incidence for 1989–1994 and 5‐year breast cancer prevalence as of January 1994. The month in which the screening program started to operate in each municipality (between January 1989 and December 1997) was provided by the National Evaluation Team for Breast Cancer Screening.^11^ As the registers are not linked at the individual level before 1995, and so vital status, as well as date and cause of death, are not available before that year, we avoid selective survival bias by restricting the analysis to 138 (out of 484) municipalities where the program began in January 1995 or later (Supporting Information Fig. S1).

We selected women aged 49–63 on January 1, 1995. Those aged 49 were included, because a woman becomes eligible for screening in the year she turns 50. Those older than 63 were excluded because the number of screening invitations they received due to the extension of the program to women aged up to 75 varied (Supporting Information Fig. S2), and we could not observe this variation because we did not know the municipality‐specific launch dates of the extension.

For each woman aged 49–63 on January 1, 1995, we identified (from municipality of residence) the month and year she was first invited for screening, whether she was alive at December 31, 2011, and, if not, the cause of death.

### Statistical analyses

The study design is valid if the program rollout was uncorrelated with prerollout breast cancer mortality risk at the municipality level. To check the plausibility of this assumption, we regressed program entry date on indicators of pre‐1995 breast cancer mortality risk, which include municipality‐specific demographics, as well as breast cancer incidence and prevalence. We include both pre‐1995 incidence and prevalence, because the change in the prevalence rate is equal to the incidence rate less the mortality rate.^19^ Hence, conditional on incidence prior to 1995, pre‐1995 prevalence varies only with the number of breast cancer deaths and so controls for municipality‐level variation in breast cancer mortality prior to 1995.

We estimated the intention‐to‐treat effect of delayed (from early 1995) initial invitation for screening on breast cancer mortality risk. We created a binary indicator equal to 1 if the woman had died of breast cancer (i.e., primary cause registered as International Classification of Diseases [ICD]‐9 code 174 or ICD‐10 code 50) by December 31, 2011, and 0 if she was alive or had died from any other cause. A linear probability model was used to estimate the effect on the probability of death from breast cancer, and the estimated regression coefficients were multiplied by 100,000 to show the impact on the number of deaths over the 17‐year period per 100,000 women initially aged 49–63.

In a first model, we used all municipalities that entered the program in 1995–1997 and regressed the mortality indicator on a series of binary variables that identified the 6‐month interval during which the program started to operate in the municipality in which each woman was resident at the start of 1995. This estimates the intention‐to‐treat effect of delayed initial screening invitation on breast cancer mortality risk. However, it does not isolate the effect of the receipt of one additional screening invitation over the entire period during which a woman is eligible for the program. Compared to women first invited in 1995, those invited in 1996 have received the same number of screening invitations in even‐numbered calendar years (1998, 2000, etc.) and one more in odd‐numbered years (1999, 2001, etc.). For this reason, we estimate a second model in which we discard municipalities that entered the program in 1996 and regressed the mortality indicator on a single binary variable that distinguished access to the program in 1997 from entry in 1995. In every year throughout our follow‐up period, women first invited in 1995 have received exactly one more screening invitation than women first invited in 1997, and so comparison of these groups identifies the effect of an additional invitation.

We used the second, more parsimonious specification to conduct sensitivity and validity analyses. Model specification was based on the “post‐double‐selection” method.^20^ We considered municipality‐specific demographics, as well as breast cancer incidence and prevalence, as potential controls, but in the models used to obtain the estimates presented, we only controlled for those variables that were significantly correlated either with breast cancer mortality from 1995 to 2011 or with the date screening started during this period (Supporting Information Table S3 confirms robustness to other model specification choices). All models controlled for age in January 1995 in years (49 reference category, binary variables for 50 and 51 to 63).

Sensitivity and validity analyses included controlling for province fixed effects, restricting to women who did not move between municipalities, replicating the analysis on women aged 72–77 in January 1995, controlling for initial screening uptake, and estimating by logistic rather than least squares regression.

We further estimated the effect of one additional biennial screening invitation on all‐cause mortality by defining a binary indicator of death from any cause between January 1995 and December 2011 and modeling this using the “post‐double‐selection” method (Supporting Information Table S6 shows estimates from other model specification choices).^20^

Standard errors were clustered over women living in the same municipality at the time of the first invitation. All hypotheses tests were against a two‐sided alternative, and a *p* value of less than 0.05 was taken to indicate statistical significance. All computations were done using Stata® version 14.

### Ethics approval, consent to participate, and data availability

This research did not have to undergo a medical ethical review according to the Dutch Medical Research Involving Human Subjects Act, because the individuals were not subjected to procedures or were required to follow rules of behavior. For the same reason, consent to participate was not required for our study.

The data that support the findings of our study derive from three sources. The majority of the data were provided by Statistics Netherlands via a Remote Access facility (GBAADRESBUS 1995–2011 and DO 1995–2011) and are available from Statistics Netherlands via https://www.cbs.nl/en-gb/our-services/customised-services-microdata/microdata-conducting-your-own-research to authorized institutions on payment of a fee. These data from Statistics Netherlands were linked to data of the Netherlands Cancer Registry (available from the Netherlands Comprehensive Cancer Organization via http://www.cijfersoverkanker.nl/data-request-58.html) and to data on program implementation dates and participation rates (available from the National Evaluation Team for Breast Cancer Screening https://www.erasmusmc.nl/public-health/research-education/screening-evaluation/?lang=en). As stipulated in the data agreement, Statistics Netherlands has the right to preview the findings of this project prior to publication to ensure that privacy sensitive, individual‐specific information is not revealed.

## Results

### Rollout of the screening program

Of the 138 municipalities that entered the screening program after 1994, 33 began screening in the first half of 1995 and 7 did not start screening until the second half of 1997 (Table 1). The size of the female population per municipality (11,606, on average) did not differ significantly across municipalities categorized by the 6‐month period in which each entered the program (Table 1). Neither did the percentage of female inhabitants who were age‐eligible for screening (19%, on average). Prior to the program operating in any of these municipalities, annual breast cancer incidence, which averaged 117 per 100,000 females of all ages in the period 1989–1994, also did not differ significantly by date of program entry. The 5‐year breast cancer prevalence (449 per 100,000 females in 1994, on average) did differ significantly (*p* = 0.03). However, multivariate analyses revealed that date of program entry was not significantly associated (individually or jointly) with preprogram prevalence, incidence, size of female population, and its share in the screening‐eligible age range (Supporting Information Tables S1 and S2 and Figs. S3 and S4). As these factors were also uncorrelated with breast cancer mortality between 1995 and 2011 (Supporting Information Table S3), they were excluded from the regression used to estimate the program impact on mortality.

**Table 1:** Preprogram characteristics of municipalities by date of entry to the screening program^1^

Date of program entry	Number of municipalities (*N* = 138)	Female population, 1989–1994^2^	Percentage of females aged 50–69, 1989–1994^3^	Breast cancer incidence, 1989–1994^4^	Five‐year breast cancer prevalence, January 1994^5^	Percentage of females aged 50–69 screened at first invitation^6^
January to June 1995	33	11,045 ± 16,349	19 ± 2	112 ± 23	419 ± 104	81 ± 5
July to December 1995	34	10,887 ± 10,785	19 ± 3	117 ± 25	438 ± 100	81 ± 3
January to June 1996	33	12,968 ± 13,851	19 ± 2	120 ± 37	487 ± 111	81 ± 4
July to December 1996	19	13,721 ± 17,217	20 ± 3	130 ± 29	480 ± 87	80 ± 6
January to June 1997	12	10,175 ± 10,574	19 ± 3	113 ± 43	460 ± 164	82 ± 5
July to December 1997	7	8,044 ± 4,601	19 ± 4	102 ± 29	373 ± 115	79 ± 6
*p* value^7^		0.95	0.47	0.31	0.03	0.89
All municipalities	138	11,606 ± 13,645	19.0 ± 2.0	117 ± 30	449 ± 111	81 ± 5

1 Plus–minus values are means ± SD. Only municipalities that started organized screening between January 1995 and December 1997.

2 The number of females in a municipality averaged over the period 1989–1994.

3 Percentage of the female population in a municipality who is aged 50–69 and so eligible for screening, averaged over the period 1989–1994.

4 Municipality‐specific median over the period 1989–1994 of the annual number of newly diagnosed breast cancer cases per 100,000 female inhabitants.

5 The number of cancer patients who were diagnosed with breast cancer in the 5 years preceding January 1994 and still alive at that time per 100,000 female inhabitants of a municipality.

6 Data available for only 133 municipalities.

7 *p* values are for the Kruskal‐Wallis test of no difference by date of program entry.

### Subjects

In a population of 256,712 women who in January 1995 were aged 49–63 and resident in the 138 municipalities where screening had not yet started by that date, there were 3,604 (1.4%) breast cancer deaths and 38,341 (14.9%) deaths in total over the 17 years to December 2011. Mean (±standard deviation [SD]) age in January 1995 was 55.6 (±4.4) years. For the estimation of the impact of one additional screening invitation, we drop all women first invited in 1996. This leaves us with 148,920 women in 86 municipalities who experienced identical cumulative breast cancer (1.4%) and all‐cause mortality rates (14.9%) over the 17 years to December 2011.

### Effects on breast cancer mortality

The top panel of Table 2 shows the effects of delayed access to the screening program on cumulative breast cancer mortality between 1995 and 2011. It shows (rescaled) estimates from a regression of the binary indicator of breast cancer death on binary indicators of the half‐year interval in which each woman's municipality entered the program. For every 100,000 women aged 49 who lived in municipalities where the program was implemented between January and June 1995, we estimated that 1,138 had died from breast cancer by the end of 2011 (95% confidence interval [CI], 952 to 1,324; *p* < 0.001). There was no significant (statistically or substantively) effect on breast cancer mortality of accessing the program up to 18 months after those covered from the first half of 1995. Once the delay lengthened to 2 years, the estimated effect increased greatly in magnitude to reach a significant 163 additional deaths per 100,000 women over the 17 year period. Gaining access to the program 2 years earlier is estimated to have reduced the risk of dying from breast cancer within 17 years by 10.4% (163/(1,138 + 163 + 266)) for women aged 56 (mean age; Supporting Information Table S4). The point estimate of the effect increased further, although not significantly, when the delay reached 2.5 years.

**Table 2:** Association between date of entry to screening program and cumulative breast cancer mortality 1995–2011

Date of program access	Number of breast cancer deaths per 100,000 (95% CI)^1^	Difference in deaths per 100,000 from number in reference (95% CI)^2^	*p* value
Effects of delayed access to the screening program (6 month indicators; *N =* 256,712)^3^			
January to June 1995 (reference)	1,138 (952 to 1,324)		
July to December 1995		35 (−104 to 174)	0.62
January to June 1996		−44 (−161 to 74))	0.46
July to December 1996		10 (−137 to 157)	0.90
January to June 1997		163 (21 to 306)	0.03
July to December 1997		186 (33 to 339)	0.02
Effect of one additional biennial screening invitation (1997 indicator; *N =* 148,920)^4^			
1995 (reference)	1,116 (901 to 1,332)		
1997		154 (40 to 267)	0.009

1 Scaled model constant indicating estimated mortality in the reference group aged 49 and with access to the screening program from the period indicated in left‐hand column.

2 Scaled coefficient on indicator of period in which screening program started to operate in municipality.

3 Data for women aged 49–63 in January 1995 in 138 municipalities where the screening program was implemented between January 1995 and December 1997. Estimates are scaled coefficients from linear probability models with the dependent variable a binary indicator of having died from breast cancer between 1995 and 2011. The models include a (series of) binary indicator(s) of the period in which the screening program started to operate in the municipality of residence and yearly indicators of age in January 1995 (49 [reference], dummies for 50, 51, to 63). Statistical inference accounts for clustering among women in a municipality at its entry to the program.

4 Data for women aged 49–63 in January 1995 in 86 municipalities where the screening program was implemented between January and December 1995 and between January and December1997. Estimates are scaled coefficients from linear probability models with the dependent variable a binary indicator of having died from breast cancer between 1995 and 2011. The models include a (series of) binary indicator(s) of the period in which the screening program started to operate in the municipality of residence and indicators of age in January 1995 (49 [reference], dummies for 50, 51, to 63). Statistical inference accounts for clustering among women in a municipality at its entry to the program.

The bottom panel of Table 2 shows the estimate of the mortality effect of one additional biennial screening invitation by excluding municipalities that got access to the program in 1996. Municipalities that implemented the program in 1997 are estimated to have experienced an additional 154 breast cancer deaths per 100,000 women by the end of 2011 (95% CI, 40 to 267; *p* = 0.009) compared to those that entered in 1995. For a women aged 56 (mean age in our sample), this corresponds to a 9.6% (154/(1,116 + 154 + 339)) reduction in the risk of breast cancer death for those who gained access to screening in 1995 and so received one more invitation for screening over the years they were age‐eligible for the program (Supporting Information Table S3).

**Figure 1:**
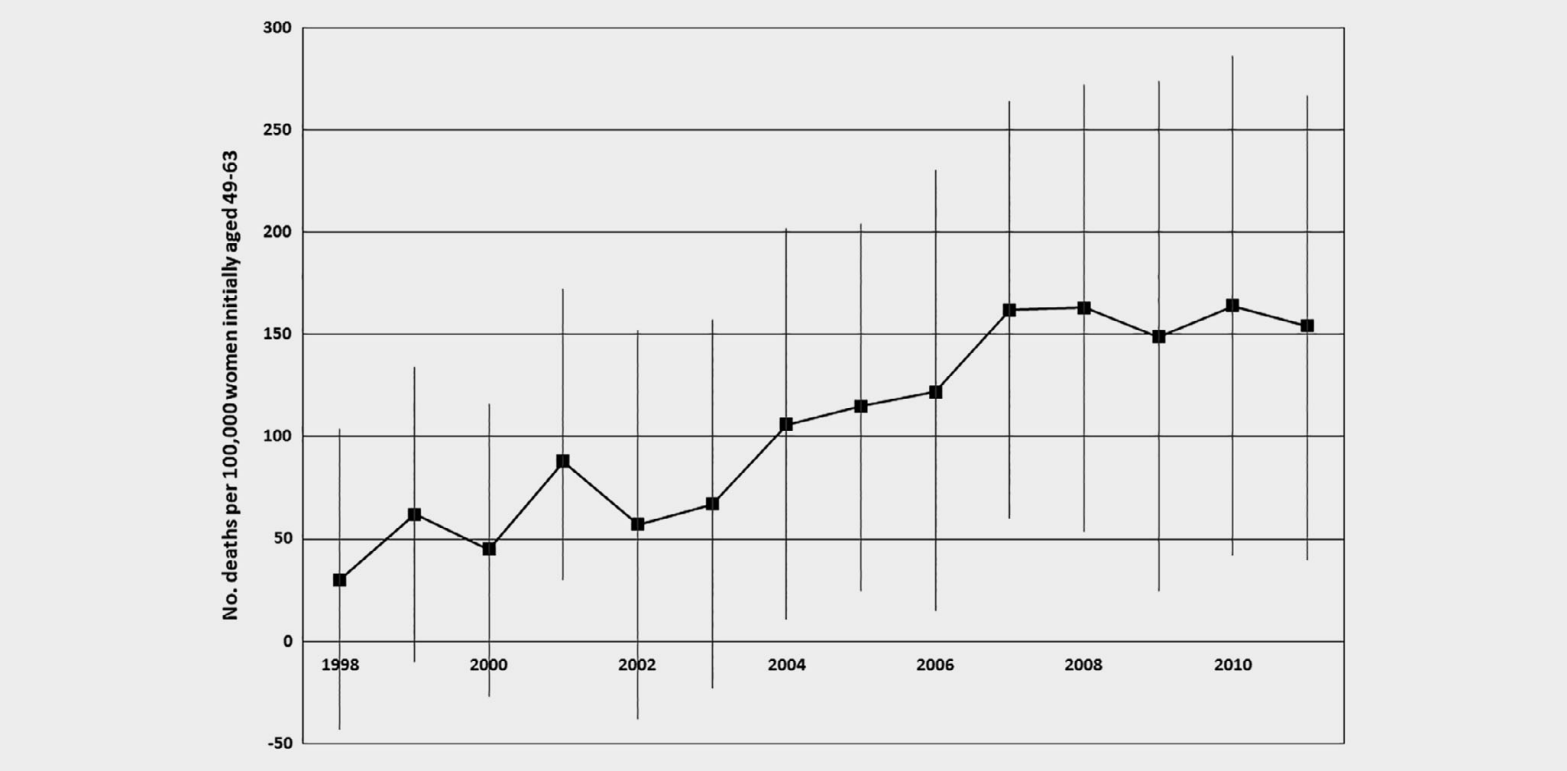
Effect of one additional biennial screening invitation on cumulative breast cancer mortality to different endpoints. The figure shows the estimated effect of getting access to the screening program in 1997 rather than 1995 on the number of breast cancer deaths from January 1995 to the end of each year indicated per 100,000 women who were aged 49–63 in January 1995. Estimates are scaled coefficients on the binary indicator of year of program entry in linear probability models with the dependent variable a binary indicator of having died from breast cancer between January 1995 and the end of the respective year. All models include yearly indicators of age in January 1995, that is, 49 (reference) and dummies for 50, 51, to 63. Error bars show 95% confidence intervals computed allowing for clustering at the municipality level.

Figure 1 shows how the estimated number of excess breast cancer deaths that arose from one additional biennial screening invitation accumulated over the follow‐up period. The point estimate for 1998 indicates that between 1995 and 1998, there were 30 extra deaths per 100,000 women aged 49–63 who gained access to screening in 1997 compared to those getting access in 1995, although the estimate is insignificant. From 2004, the effect of entry to the program in 1997 versus 1995 on the accumulated number of deaths is consistently significant. The number of excess deaths increased until 2007 and then leveled off, indicating that 10 years after the differential exposure to screening it ceased to impact on breast cancer mortality.

### Sensitivity and validity checks

The analyses reported in Table 3 demonstrate robustness of the estimates and support the validity of the study design to estimate the effect of one additional biennial screening invitation. To allow for province‐level differences in breast cancer mortality (“province fixed effects”), we had to drop 50 municipalities in three provinces where the program had been fully implemented by 1997. Although losing 47% of the sample of women reduced statistical precision leading to a marginally insignificant estimate, the estimated effect of an additional screening invitation is contained in the 95% CI reported in Table 2. Then, controlling for province fixed effects increased the estimate but not substantially. Restricting the sample to women who did not move between municipalities reduced the estimate to some degree but again not significantly. When we applied the study design to women aged 72–77 in January 1995 who were not eligible for screening, we found no effect. This indicates that breast cancer mortality in this population was unrelated to obtaining access to the program in 1995 versus 1997 and supports the assumption that background risk in the eligible population did not vary systematically across municipalities that entered at different times.

**Table 3:** Effect of one additional biennial screening invitation on cumulative mortality—sensitivity and validity checks^1^

Check	Included confounders	Effect of program access in 1997 versus 1995 on deaths per 100,000 (95% CI)	*p* value
Effect on breast cancer mortality, eligible women aged 49–63 in January 1995			
Control for province fixed effects (*N* = 69,254)^2^			
No	Age January 1995	114 (−40 to 268)	0.14
Yes	Age January 1995	139 (24 to 254)	0.02
Exclude women moving out of/from municipality in 1995–1997 (*N* = 143,424)	Age January 1995	126 (13 to 238)	0.03
“Placebo effect” on breast cancer mortality, ineligible women aged 72–77 in January 1995 (*N* = 33,081)	Age January 1995	−101 (−544 to 342)	0.65
Effect on all‐cause mortality, eligible women aged 49–63 in January 1995 (*N* = 148,920)^3^	Age January 1995; female population size (1989–1994)	392 (−621 to 1,406)	0.44

1 Estimates are scaled coefficients on binary indicator of program entry in 1997 rather than in 1995 from linear probability models with the dependent variable a binary indicator of having died between 1995 and 2011. All regressions include age in January 1995 in years (49 [reference category], dummies for 50, 51, to 63, except in placebo test to 72 [reference category], 73, 74, 75, 76, 77). Statistical inference accounts for clustering among women in a municipality at its entry to the program.

2 Municipalities in the three provinces where screening was already fully implemented by the beginning of 1997 are dropped.

3 Female population size is the number of females in a municipality averaged over the 1989–1994 period.

Analyses conducted using women in a subset of 83 municipalities with requisite data confirmed that the timing of program rollout was also uncorrelated with screening uptake at the first invitation (Table 1 and Supporting Information Fig. S5 and Tables S1 and S2). Controlling for this participation rate had little impact on the estimated effect of one additional biennial screening invitation (Supporting Information Table S5).

The point estimate indicates that access to the program in 1997, as opposed to 1995, raised all‐cause mortality but the estimate is very imprecise and not statistically significant. Although we could not establish that the geographical rollout of the program by municipality was unrelated to all‐cause mortality risk prior to 1995, we were able to confirm that rollout in 1997 was unrelated to all‐cause mortality rates in 1995 (discussion in Supporting Information).

Estimation by logistic regression rather than least squares produced no appreciable change in the estimates (Supporting Information Tables S1–S6).

Some additional sensitivity checks are reported in the Supporting Information.

## Discussion

Our study found that the Dutch screening mammography program reduced breast cancer mortality. This has been shown before, but more importantly, this is the first and only estimate of the effect of one additional screening invitation in the literature. High‐quality data on screening invitation and on individual mortality available in the Netherlands made this analysis possible. Women initially aged 49–63 who received one more invitation for screening over the entire time they were eligible (by age) for the program were estimated to experience 154 fewer breast cancer deaths per 100,000 over 17 years. This is equivalent to a 9.6% reduction in the cumulative risk of dying from breast cancer from one additional screening invitation over the eligible age range. This estimate is much lower than the 23% reduction the International Agency for Research on Cancer estimated by averaging effects of different lengths of exposure to screening.^5^ It is substantially smaller than an estimate of the impact of the Dutch breast cancer screening program based on a before‐and‐after study,^11^ but it is not at odds with the Two‐County Trial where a difference of 2.5 screens during 7 years did lead to a 27 to 31% reduction in breast cancer mortality after 29 years.^21^ Case‐control evaluations of the Dutch program^15^, ^16^ produced larger estimates of the mortality effect, because they estimated the impact of the screen(s) (not invitation) preceding diagnosis, which are obviously the most effective.

Nationwide advances in breast cancer treatment do not confound our estimates as we follow a single cohort of women over time. However, if there were geographical variation in quality of breast cancer treatment that is correlated with the spread of the program, then it could lead to bias. Our sensitivity checks and the pattern in Figure 1 suggest that this is unlikely, but it cannot be ruled out *a priori*. Sankatsing et al.^22^ recently focused on differences among early (1987–1992), intermediate (1993–1994), and late screening adopting municipalities (1995–1997) and found similar screening‐attributable reductions in breast cancer mortality, suggesting that the screening (invitation) effect is not confounded by the increased availability of adjuvant treatment between early and late adopters. This was further corroborated by not detecting an effect on older women not eligible for screening (Table 3 and discussion in Supporting Information), although finding no statistically significant effect on all‐cause mortality (Table 3) could be due to insufficient power.

Our estimate may also be smaller than earlier estimates of the impact of the Dutch breast screening program, because it was obtained from a slightly younger population (49–63 compared to, respectively, 55–74^16^, ^22^ and 50–69^11^, ^15^).

Use of administrative data covering a period of 17 years allowed us to monitor breast cancer mortality among women who received their first screening invitation at different ages but who were otherwise similar. Support for the validity of the study design was demonstrated by the fact that the program rollout was not related to preprogram breast cancer mortality risks (i.e., incidence and prevalence of breast cancer jointly). By making comparisons within a single cohort of women, we avoided relying on simple time trend analysis, comparison of different cohorts, or a combination of these study designs. These approaches do not adequately account for underlying trends in breast cancer mortality^12^, ^23^, ^24^ or they rely on strong assumptions to separate the effect of breast cancer screening access from confounding trends (discussion in Supporting Information).^11^, ^13^, ^14^, ^15^, ^16^ Advances in breast cancer awareness^25^, ^26^ did not confound our estimate, because we followed a single cohort of eligible women over time.

Our study has some limitations. One is that we have no information on opportunistic mammography screening (and/or cancer treatment) in the municipalities prior to the implementation of screening. Therefore, we cannot rule out *a priori* that opportunistic screening uptake was systematically related to the rollout of the program, even though opportunistic screening is and was uncommon in the Netherlands.^27^ Opportunistic screening and overdiagnosis can also weaken the correlation of breast cancer mortality with each of incidence and prevalence prior to 1995,^27^ but that does not invalidate using incidence and prevalence together to control for pre‐1995 breast cancer mortality as change in prevalence equals incidence minus mortality.^19^ Another limitation is that we had no information on advanced‐stage breast cancer diagnoses among the women in our cohort and we cannot confirm at the same individual level that the effect we find of one additional screening invitation on breast cancer deaths arises primarily by reducing the incidence of advanced‐stage breast cancers, which would be the most likely mechanism.^27^, ^28^, ^29^ Other mechanisms sometimes claimed to explain changes in mortality after the introduction of screening induced change in the attribution of deaths to breast cancer^30^, ^31^, ^32^, ^33^ and improvements in patient management. The high degree of reliability of the Dutch cause of death statistics,^34^ the positive but insignificant point estimate for an effect on all‐cause mortality, and the robustness of the estimates to allowing for province‐fixed effects all cast some doubt on the relevance of these potential mechanisms to the interpretation of our results (discussion in Supporting Information). That said, we are not able to falsify these mechanisms with the data available. A final limitation concerns the evolution of breast cancer deaths among the cohort of women who got access to the program in 1996. We exclude these women when estimating the impact of one additional biennial screening invitation (bottom panel Table 2) because the number of invitations they received compared to the number of invitations received by women who were first invited in 1995 (and in 1997) varies over the follow‐up period. Although confidence in the validity of the research design is bolstered by the robustness of the estimated effect of an additional screening invitation to various sensitivity checks and by the absence of any effect when the empirical strategy is applied to a cohort of older women who were not exposed to the screening program, failing to find that breast cancer mortality increases significantly and continuously with increases in the length of delay to program entry over the first 2 years examined (1995–1996; top panel Table 2) does not lend further support to the effectiveness of screening. However, comparison made between municipalities entering the program at different times in 1995 and 1996 do not separate the pure effect of delayed entry from that of receiving an additional screen.

## Conclusions

We used high‐quality data to produce the first estimate of the effect of one additional invitation for screening mammography on breast cancer mortality and deliver evidence in favor of the effectiveness of such screening. We estimate that one extra invitation for screening reduced breast cancer mortality by 10%.

## Supporting information


**APPENDIX S1**: MATERIAL FOR ONLINE PUBLICATION
