# Supplementary material for: Effect of screening mammography on breast cancer mortality: Quasi‐experimental evidence from rollout of the Dutch population‐based program with 17‐year follow‐up of a cohort
Source: Int J Cancer. 2019 Aug 7;146(8):2201–8. doi: 10.1002/ijc.32584 (PMC7065105; doi:10.1002/ijc.32584)
Supplement: Supplementary file 1 — APPENDIX S1: MATERIAL FOR ONLINE PUBLICATION [file IJC-146-2201-s001.docx]

**APPENDIX MATERIAL FOR ONLINE PUBLICATION**

**Effect of screening mammography on breast-cancer mortality: Quasi-experimental evidence from rollout of the Dutch population-based program with 17-year follow-up of a cohort.**

Tom Van Ourti, Owen O’Donnell, Hale Koç, Jacques Fracheboud, Harry de Koning

**METHODS**

*Screening program – further details*

Women who did not respond to the screening invitation were sent a reminder after 2-3 months. Incomplete take-up does not invalidate the study design since we showed that the rollout of the screening program was unrelated to the participation rate at the first invitation (Fig. S5, Table S1, S2).

During the period (1995-97) of variation in screening exposure used in the study, the primary test offered was X-ray mammography. Since the second half of 2004, digital screening began to be offered. By 2010, 94 percent of the screening examinations were digital. We did not have information to establish whether the spread of digital screening since 2004 was related to the geographic rollout of the program between 1995 and 1997. We found that, prior to digital screening being available, earlier receipt of a first invitation caused a statistically significant reduction in breast-cancer mortality.

*Data and study population – further details*

We used a key provided by The National Evaluation Team for Breast Cancer Screening to link each woman’s zipcode recorded in the administrative data to the launch date of the screening program in every municipality between 1995 and 1997.^1^ This key identifies the launch dates in municipalities that existed in 1999. Out of 197 municipalities that started organized screening in 1995 and after, 54 had merged with bigger municipalities by 1999. We could not use the key to identify the launch date in these municipalities. We dealt with this by defining the date of first invitation for a woman living in municipality A that was merged into municipality B sometime before 1999 according to the program launch date in B. This is unlikely to have caused much bias because municipalities usually merged with adjacent municipalities with similar launch dates. We dropped 5 municipalities for which the Dutch Cancer Registry^2^ could not provide breast cancer incidence/prevalence data. This left us with 138 (=197-54-5) municipalities in the dataset.

Women who, after January 1995, moved into municipalities that started screening after that date were not included in the study. From a pool of 263,777 women who on January 1, 1995 were aged 49-63 and lived in municipalities that started screening after that date, we dropped 4,803 who emigrated from the Netherlands and another 82 who, before receiving their first invitation, moved to municipalities for which we do not know the program start date. We also excluded 1,793 women who moved to municipalities that started organized screening before 1995 and who got their first invitation there. Finally, we excluded 387 women who, due to moving frequently between municipalities, received their first invitation after December 1997. For 1,380 women who were alive in January 1995 but who died before the program started in their municipality, we attributed the program entry date of their municipality of residence in January 1995. The analyses in the bottom panel of Table 2 did not consider women living in municipalities where screening was introduced in calendar year 1996, leading to a sample of 148,920 observations.

*Statistical Analyses – further details*

The estimates presented in the top panel of Table 2 (Six month indicators) were derived from the following model, $Y_{im}=\alpha+\sum_{j=1}^{5} \beta_{j}START_{m}^{j}+\sum_{k=50}^{63} \gamma_{k}AGE_{i}^{k}+\varepsilon_{im}$ *, w*here *Y_im_* is a binary variable equal to 1 if woman *i* located in municipality *m* died from breast cancer by the end of 2011, and equal to 0 otherwise, i.e. alive or died from another cause. $START_{m}^{j}$ is a binary variable equal to 1 if during the period 1995-97 the women was located in a municipality *m* in which the screening program started to operate in the 6-month period indicated by *j* (j=1 corresponds to July-December 1995, …, j=5 corresponds to July-December 1997 and January-June 1995 is the reference) and equal to 0 otherwise. $AGE_{i}^{k}$ is a binary variable that is equal to 1 if the woman’s age in January 1995 was equal to *k* (49 is the reference category). It was appropriate to condition on age because it is the main risk factor for breast cancer and it determines the number of screening invitations received after entry to the program (see Fig. S2). Conditional on age, the number of invitations received varied only by municipality. *ε_im_* is a random error term representing unobserved determinants of breast cancer mortality that may vary over women and municipalities.

The estimates presented in the bottom panel of Table 2 (1997 indicator) were derived after removing all women that lived in municipalities that got access to screening in 1996; and from imposing an alternative specification in which the six month indicators were replaced by a single indicator (*START_m_*) of being located in a municipality where the program started in 1997 $\left(START_{m}=1\right)$ rather than in 1995 $\left(START_{m}=0\right)$: $Y_{im}=\alpha+\beta START_{m}+\sum_{k=50}^{63} \gamma_{k}AGE_{i}^{k}+\varepsilon_{im}$. Exclusion of women that got access in 1996 allows consistent estimation of the effect of one additional screening invitation since women invited in 1995, as compared to those invited in 1996, have the same number of screening invitations in even calendar years (1998, 2000, …) and one more in uneven years (1999, 2001, …). Therefore, these women cannot be used to consistently estimate the effect of one additional screening invitation.

If the rollout created variation in the date of entry to the program was not correlated with breast-cancer mortality risk, then $\beta$ is the causal effect on the probability of dying from breast cancer as a result of one additional biennial screening invitation.

To check whether the rollout created variation in entry to the program was exogenous to background breast-cancer mortality risk, we used municipality level data to conduct a number of tests for association between measures of the time of entry and indicators of pre-program risk. In the absence of data on breast-cancer mortality prior to 1995, the pre-program crude breast cancer incidence and prevalence rates were used as proxies. Since incidence counts the number of new breast cancer cases and prevalence counts the number of survivors, conditional on incidence, prevalence varies only with the number of breast-cancer deaths.^3^ Thus, controlling for both breast cancer incidence and prevalence prior to 1995 is an indirect way of controlling for pre-1995 breast cancer mortality. We also checked whether rollout was correlated with the female population size and the fraction in the screening eligible age range.

The first assessment of association involved separately testing whether female population size, percentage of females in the eligible age range, incidence and prevalence each differed across municipalities grouped by the 6-month period of entry to the program. P-values of the respective Kruskal-Wallis tests are reported in Table 1. More consistent with our approach, we regressed measures of program entry date on various combinations of the four characteristics. For example, using the single measure that distinguished entry in 1997 from entry in 1995 (and excluding municipalities where the program arrived in 1996), we estimated:

*START_m_ = α_0_ + α_1_Incidence_m_ + α_2_Prevalence_m_ + α_3_Population_m_ + α_4_Target_m_ + u_m_* , (1)

where *Incidence_m_* is the municipality-specific median over the period 1989-1994 of the annual number of newly diagnosed breast-cancer cases per 100,000 female inhabitants, *Prevalence_m_* is the number of cancer patients who were diagnosed with breast cancer in the 5 years preceding January 1994 and still alive at that time per 100,000 female inhabitants of a municipality, *Population_m_* is the average size of the female population in the period 1989-1994 and *Target_m_* is the percentage of the female population that was aged 50-69 and so eligible for screening averaged over the period 1989-1994. Estimates of alternative specifications of this model are presented in Table S2.

Similar ordered probit models, that also included municipalities where the program entered in 1996, were estimated with the dependent variable defined as a categorical measure of the 6-month period in which a municipality entered the program. These estimates are presented in Table S1. We also estimated similar models with the dependent variable defined as the number of months from January 1995 until entry to the program.

Absence of correlation between the program entry date and indicators of pre-program breast-cancer mortality risk is insufficient grounds for excluding these potential confounders from the model that was used to estimate the program effect. Inference for the intervention effect would be invalid if the potential confounders were correlated with breast-cancer mortality in the period 1995-2011 conditional on date of program entry.^4^ We used the “post-double-selection” method to choose the model specification: a covariate was included if it was significantly correlated either with the timing of program entry or with breast-cancer mortality given the date of program entry.^4^

All estimates presented in the paper were obtained from linear probability models for the binary indicator of dying from breast cancer that were estimated by ordinary least squares. Rather than show the estimated effect on the probability of breast-cancer death, we multiplied the coefficient by 100,000 to obtain the estimated effect on the number of breast-cancer deaths per 100,000 women initially aged 49-63. We show in Table S3-S6 below that the findings are highly robust to using logistic regression to estimate average partial effects using the margins command in Stata®. In all models, standard errors are clustered over women living in the same municipality at the time of the first invitation using the cluster() option in Stata®.

**RESULTS**

*Rollout of the screening program*

Table 1 in the paper shows that there were no significant differences in pre-program female population size, the percentage of females in the eligible age range and breast-cancer incidence across municipalities grouped by date of entry to the program. There was a significant difference in pre-program prevalence. Consistent results were obtained using an F-test that is more powerful than the Kruskal-Wallis test presented in Table 1 but imposes the assumption of normality. P-values from the F-tests (robust to heteroscedasticity) were 0.55, 0.38, 0.19 and 0.03 for population size, fraction 50-69, incidence and prevalence respectively. The significant difference found in pre-program breast-cancer prevalence may arouse doubt about validity of the study design. However, prevalence is only a proxy for mortality conditional on incidence. Rather than examine its simple correlation with program entry date, it is more appropriate to determine whether this potential confounder, and each of the others, is associated with the intervention variable conditional on other controls, and whether it is associated with the outcome conditional on the intervention and controls. We performed these checks and present the results in this and the next sub-sections.

Table S1 presents estimates from various specifications of an ordered probit model of a categorical dependent variable indicating the 6-month period (i.e. first half of 1995, …, second half of 1997) in which a municipality joined the program. Table S2 presents estimates from various specifications of the linear probability model, given in equation (1), of the binary indicator of entering the screening program in 1997 as opposed to 1995. Estimates of logistic regression models of the same binary variable are also given in Table S2. The findings from all these models are highly consistent, they show no association between date of program entry and the four independent variables in (1). The same conclusion was reached on the basis of linear regression models of the number of months that passed from January 1995 before a municipality entered the program. These results are not presented but are available from the authors on request.

The most important result in Table S1 is that the probability of entering the program in any particular 6-month period was jointly unrelated to both breast-cancer incidence and prevalence prior to 1995 (columns I). Similarly, Table S2 shows that the probability of entering in 1997, as opposed to 1995, was not related to previous incidence and prevalence. Since prevalence varies only with mortality given incidence, this implies that the timing of entry was not related to pre-program breast-cancer mortality. This finding is in line with Fig. S3 and S4. Column II of Table S1 shows that there was no correlation between the 6-month period in which the municipality entered the program and both the pre-program size of the female population and the share of that population in the eligible age range. Table S2 shows independence of the probability of entering in 1997 (versus 1995) from population size and fraction in the eligible age range.

From the National Evaluation Team for Breast Cancer Screening, we obtained data on the percentage of women who accepted the invitation for screening when the program was first launched in their municipality.^1^ This information was unavailable for 5 municipalities. In the remaining municipalities, the average participation rate was 81%. While there is some variation in take-up across municipalities, it is clear from Table 1 and Fig. S5 that it was not correlated with the date of entry to the program. Consistent with this, the regression estimates in column III of Tables S1 and S2 show that the 6-month period of program entry and the probability of entry in 1997 versus 1995 respectively did not vary with the initial participation rate. Column IV of Table S1 (S2) reveals that the 6-month period of program entry (entry in 1997) was not significantly correlated with any of the covariates (individually or jointly).

For sub-sets of municipalities with available data, we have also confirmed that the program launch date was not correlated with average household income in 2005 (N=122) and with the female labor force participation rate in the period 1996-1998 (N=82). These results are available on request.

*Robustness of estimated effect of one additional biennial screening invitation on breast-cancer mortality*

The ‘post-double selection’ method^4^ of model specification that we followed involved checking whether potential confounders were uncorrelated with breast-cancer mortality, conditional on date of program entry, in addition to being uncorrelated with program entry. Column Ia of Table S3 and Ia of Table S4 show the full models from which the estimates presented in Table 2 were taken. The significance of the age category variables justifies their inclusion. Column Ib of Tables S3 and S4 demonstrate that the estimated effect is highly robust to using logistic regression rather the linear probability model. The subsequent columns of Table S3 reveal that controlling for pre-program breast-cancer incidence and prevalence resulted in only a very marginal increase in the point estimate of the effect of one additional screening invitation. Controlling for female population size and the fraction of females aged 50-69 increased the estimated effect with the same small margin. However, even when both sets of controls were included (Columns IVa-b), the estimated effect was identical to that obtained from the most parsimonious specification.

Apart from the woman’s age, none of the covariates considered as potential confounders was ever even close to being significantly associated with the probability of dying of breast cancer. Together with the results presented in Table S1 and S2, this finding justifies the specification that was used to produce the estimates presented in the paper that controlled only for age.

*Table 3: Sensitivity and validity checks*

Province fixed effects

*Motivation*: We identified the program effect from variation in the date of entry to it across municipalities. The results presented in Table S1 and S2 are reassuring with respect to the assumption that rollout of the program was exogenous to breast-cancer mortality risks. But one cannot rule out unobservable determinants of the risk that varied systematically across municipalities distinguished by their dates of program entry. Eliminating the influence of any such correlated unobservables by allowing for municipality fixed effects was not possible because they would be perfectly collinear with the date of program implementation in each municipality. However, we could allow for time invariant unobservable determinants of breast-cancer mortality at the province level that were potentially correlated with the timing of program entry. An example would be variation in treatment quality across provinces, although the universal and comprehensive health care coverage in the Netherlands should limit the scope for such inequality.

*Method & results*: The sample size in the top panel of Table 3 (N=69,254) is considerably smaller than that in Table 2 because allowing for province fixed effects necessitated dropping 50 municipalities in 3 provinces where the screening program had been fully implemented by the beginning of 1997. With this restricted sample, estimates of average partial effects from logistic regression models are extremely close to linear probability model estimates obtained with and without control for province fixed effects (see Table S5 columns I and II).

Excluding women who moved between municipalities

*Motivation*: In defining our intervention variables, we categorized a woman according to the municipality in which she was resident at the time she first had an opportunity to be invited for screening. For example, a woman initially resident in municipality A where the program started in June 1995 and who moved in October 1995 to municipality B where the program started in June 1996, was classified as receiving her first invitation in June 1995. Another woman, who moved from B to A in April 1995 was also classified as receiving her first invitation in June 1995.

It seems highly unlikely that any woman would have moved between municipalities in order to access screening mammography. But since it cannot be ruled out entirely, we conducted a sensitivity analysis that involved restricting the sample to women who did not move between municipalities over the period 1995-97.

*Results*: The insignificant reduction in the estimated number of breast-cancer deaths attributed to receiving a first invitation in 1997 rather than 1995 is in the opposite direction to that expected if women at high risk of breast cancer had moved to municipalities that entered the program earlier. The estimate obtained with the exclusion of women who relocated is the same irrespective of whether it was obtained from a linear probability or a logistic regression model (Table S5, column III).

‘Placebo test’

*Motivation*: If the estimated reduction in the breast-cancer mortality of screening-eligible women that is attributed to earlier access to the program was simply an artefact, then application of the same study design to a group ineligible women exposed to the same change in municipality-specific breast-cancer mortality that was not caused by the program and may, for example, have been due to improved treatment, then this would produce a significant ‘effect’ in this group that could not possibly have benefited from the program.

*Method & result*: We used women aged 72-77 in January 1995 to perform the ‘placebo test’ because not only were they ineligible for the program in its initial form but neither did they benefit from its extension in 1998 to women aged up to 75 years. We selected older women living in municipalities where screening started being offered to women aged 50-69 after 1994. The absence of any significant ‘effect’ on mortality of the older group does not appear to be simply attributable to its smaller sample size (N=33,081 compared with N=148,920), although this is one reason for the much wider confidence interval. The point estimate is negative for the older group. This is inconsistent with our estimate of 154 additional breast-cancer deaths per 100,000 women aged 49-63 being attributable to a coincidental increase in mortality in the municipalities that started operating screening in 1997 as opposed to 1995. Again, it made no difference whether we estimated the ‘effect’ in the older group using a logistic regression rather than a linear probability model (Table S5, column IV).

Effect on all-cause mortality

*Motivation*: Potential harm caused by overdiagnosis and treatment of clinically insignificant cancers motivates interest in the impact of screening mammography on all-cause mortality.^5-9^ It is also possible that competing risks lead to a differential impact on all-cause mortality as compared to breast cancer mortality after 17 years of follow-up.^10,11^ Since the (net) effect of screening (invitations) is likely to be small relative to the variability in total mortality, it is difficult to obtain a precise estimate of the impact of screening (invitation) on this outcome. While recognizing this, we attempted estimation in order to check whether there was any evidence that the negative effect of the screening program on breast-cancer mortality was offset by increased mortality due to other causes. The argument that screening changes the attribution of deaths to breast cancer^12,13,14,15^ is unlikely to have any force because the Dutch cause-of-death statistics have a high degree of reliability.^16^

*Method & result*: Since we did not have mortality data prior to 1995, we could not check the validity of the study design for estimation of the effect on all-cause mortality by examining whether the geographical spread of the screening program was related to all-cause mortality risks prior to 1995. We were able to confirm that the month-by-month rollout of the program in 1997 was unrelated to the municipality-specific all-cause mortality rate in 1995 among municipalities that started screening after 1996. While this is a less comprehensive test than those we conducted for breast-cancer mortality, the findings did not cause us to question the appropriateness of the study design for estimation of the effect on all-cause mortality.

The estimate of the effect of program entry in 1997 as opposed to 1995 on all-cause mortality presented in Table 3 was obtained from a model that controlled for the size of the female population prior to 1995, as well as age. This specification followed from application of the “post-double-selection” criterion^4^. This can be seen from the results presented in Table S6. When all covariates were included in the model (Columns IIIa-b), only the size of the female population prior to 1995 was significant. The preferred specification was therefore that given in Columns IVa-b that included only this variable.

While the point estimate of the effect of program entry in 1997 on all-cause mortality varies greatly from specification to specification, in no case does it ever come close to being statistically significant. This is the imprecision we had anticipated. The point estimate obtained from the preferred specification, and presented in Table 3, is considerably larger than the estimated effect on breast-cancer mortality. While the hypothesis that the impact on all-cause mortality was smaller than the impact on breast-cancer mortality could not be rejected, we tentatively conclude that there is no support in the data for the proposition that screening had an offsetting effect on mortality from causes other than breast cancer.

Once again, it made no difference whether effects were estimated by the linear probability model or by logistic regression (Table S6).

Additional checks not presented in Table 3

We showed in Table S1 and S2 (Columns III) that the timing of program entry was not correlated with the rate of screening uptake at the first invitation. We have confirmed that controlling for the initial take-up rate does not change the estimated effect one additional screening invitation on breast-cancer mortality. Dropping the three municipalities for which we had no data on initial uptake of screening resulted in a fall in the estimated effect of entering the program in 1997 rather than 1995 from 154 (Table 2) to 108 (Table S5, column V) but the estimated effect remains significant at the 5 percent level and within the 95 percent confidence interval of the baseline estimate. In the restricted sample, adding the initial take-up rate had little or no impact on the estimated effect (compare columns V and VI of Table S5). The estimated effect of one additional screening invitation does not appear to have been driven by differences in the uptake of the screening invitation across municipalities.

**DISCUSSION**

*Comparison with two case control studies of the Dutch breast cancer screening program*

Two case-control studies have estimated the mortality effect of the Dutch breast cancer screening program.^17,18^ These studies estimated that screening mammography lowered the relative risk of breast cancer mortality by 28 percent (1977-1991) and 65 percent (1992-2008) among women who attended either of the two screens preceding diagnosis compared to those who attended none,^17^ and by 44 percent (1995-2003) among women who attended the screen just before the diagnosis compared to those who did not.^18^ These estimates are larger than our estimate of a reduction of 9.6 percent in relative risk of breast cancer mortality. This is due to the fact that we estimated the mortality effect of receiving one extra invitation, on average, in a lifetime, while the case-control studies estimated the effect of attending the screening(s) prior to diagnosis that has (have) the greatest potential to make a difference. Moreover, it is difficult to rule out that cases differ from controls with respect to risk factors not accounted for in the model. We compare across women who differ in timing of entry to the screening program only as a consequence of their municipality of residence.

Figure S1. Geographical rollout of the Dutch breast cancer screening program between 1995 and 1997


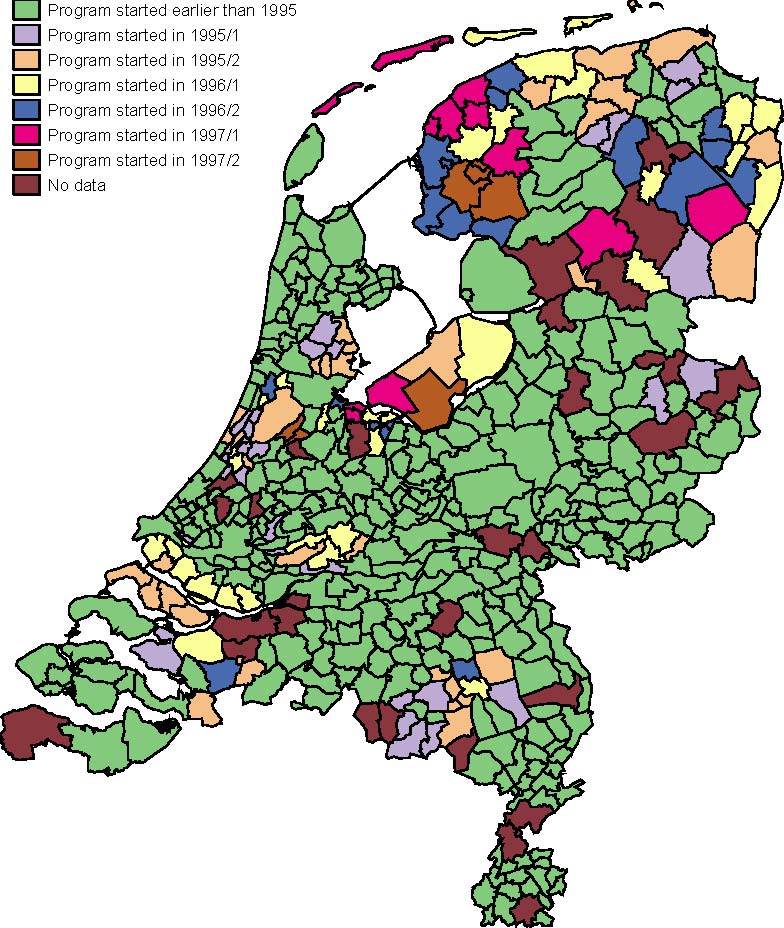


199x/1 refers to the first half of 199x; 199x/2 refers to the second half of 199x

Figure S2. Eligibility for screening mammography for women aged 49-68 in January 1995 by age and year

|  | 1995 | 1996 | 1997 | 1998 | 1999 | 2000 | 2001 | 2002 | 2003 | 2004 | 2005 | 2006 | 2007 | 2008 | 2009 | 2010 | 2011 |
| --- | --- | --- | --- | --- | --- | --- | --- | --- | --- | --- | --- | --- | --- | --- | --- | --- | --- |
| age | 49 | 50 | 51 | 52 | 53 | 54 | 55 | 56 | 57 | 58 | 59 | 60 | 61 | 62 | 63 | 64 | 65 |
|  | 50 | 51 | 52 | 53 | 54 | 55 | 56 | 57 | 58 | 59 | 60 | 61 | 62 | 63 | 64 | 65 | 66 |
|  | 51 | 52 | 53 | 54 | 55 | 56 | 57 | 58 | 59 | 60 | 61 | 62 | 63 | 64 | 65 | 66 | 67 |
|  | 52 | 53 | 54 | 55 | 56 | 57 | 58 | 59 | 60 | 61 | 62 | 63 | 64 | 65 | 66 | 67 | 68 |
|  | 53 | 54 | 55 | 56 | 57 | 58 | 59 | 60 | 61 | 62 | 63 | 64 | 65 | 66 | 67 | 68 | 69 |
|  | 54 | 55 | 56 | 57 | 58 | 59 | 60 | 61 | 62 | 63 | 64 | 65 | 66 | 67 | 68 | 69 | 70 |
|  | 55 | 56 | 57 | 58 | 59 | 60 | 61 | 62 | 63 | 64 | 65 | 66 | 67 | 68 | 69 | 70 | 71 |
|  | 56 | 57 | 58 | 59 | 60 | 61 | 62 | 63 | 64 | 65 | 66 | 67 | 68 | 69 | 70 | 71 | 72 |
|  | 57 | 58 | 59 | 60 | 61 | 62 | 63 | 64 | 65 | 66 | 67 | 68 | 69 | 70 | 71 | 72 | 73 |
|  | 58 | 59 | 60 | 61 | 62 | 63 | 64 | 65 | 66 | 67 | 68 | 69 | 70 | 71 | 72 | 73 | 74 |
|  | 59 | 60 | 61 | 62 | 63 | 64 | 65 | 66 | 67 | 68 | 69 | 70 | 71 | 72 | 73 | 74 | 75 |
|  | 60 | 61 | 62 | 63 | 64 | 65 | 66 | 67 | 68 | 69 | 70 | 71 | 72 | 73 | 74 | 75 | 76 |
|  | 61 | 62 | 63 | 64 | 65 | 66 | 67 | 68 | 69 | 70 | 71 | 72 | 73 | 74 | 75 | 76 | 77 |
|  | 62 | 63 | 64 | 65 | 66 | 67 | 68 | 69 | 70 | 71 | 72 | 73 | 74 | 75 | 76 | 77 | 78 |
|  | 63 | 64 | 65 | 66 | 67 | 68 | 69 | 70 | 71 | 72 | 73 | 74 | 75 | 76 | 77 | 78 | 79 |
|  | 64 | 65 | 66 | 67 | 68 | 69 | 70 | 71 | 72 | 73 | 74 | 75 | 76 | 77 | 78 | 79 | 80 |
|  | 65 | 66 | 67 | 68 | 69 | 70 | 71 | 72 | 73 | 74 | 75 | 76 | 77 | 78 | 79 | 80 | 81 |
|  | 66 | 67 | 68 | 69 | 70 | 71 | 72 | 73 | 74 | 75 | 76 | 77 | 78 | 79 | 80 | 81 | 82 |
|  | 67 | 68 | 69 | 70 | 71 | 72 | 73 | 74 | 75 | 76 | 77 | 78 | 79 | 80 | 81 | 82 | 83 |
|  | 68 | 69 | 70 | 71 | 72 | 73 | 74 | 75 | 76 | 77 | 78 | 79 | 80 | 81 | 82 | 83 | 84 |

Each row shows eligibility for mammography screening by year for women at the age indicated in the first column in January 1995. Green indicates there is variation in the age of receipt of the first invitation for screening due to the rollout of the program across municipalities between 1995 and 1997. Yellow indicates ages of receipt of biennial invitations after the program had been fully implemented nationwide. Red indicates ages and years in which there is variation in receipt of invitations due to the phased extension of the program to women aged up to 75. Blue indicates ages of receipt of biennial invitations after the extension to age 75 had been fully implemented nationwide. White indicates ages at which women no longer receiving an invitation.

Figure S3. Municipality pre-program breast-cancer incidence against month of entry to the program


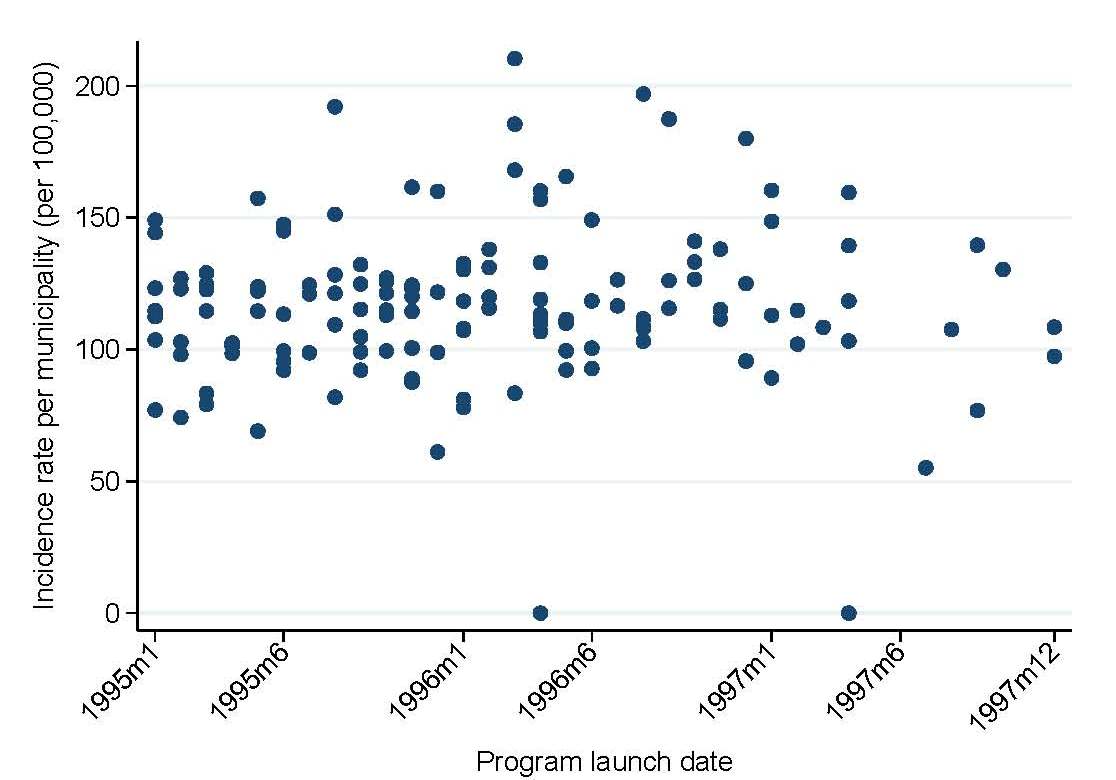


Program launch date is the month (m1 = January etc) when the program started to operate in a municipality. Incidence rate is the municipality’s median annual breast cancer incidence over the period 1989-1994.

Figure S4. Municipality pre-program breast cancer prevalence against month of entry to the program


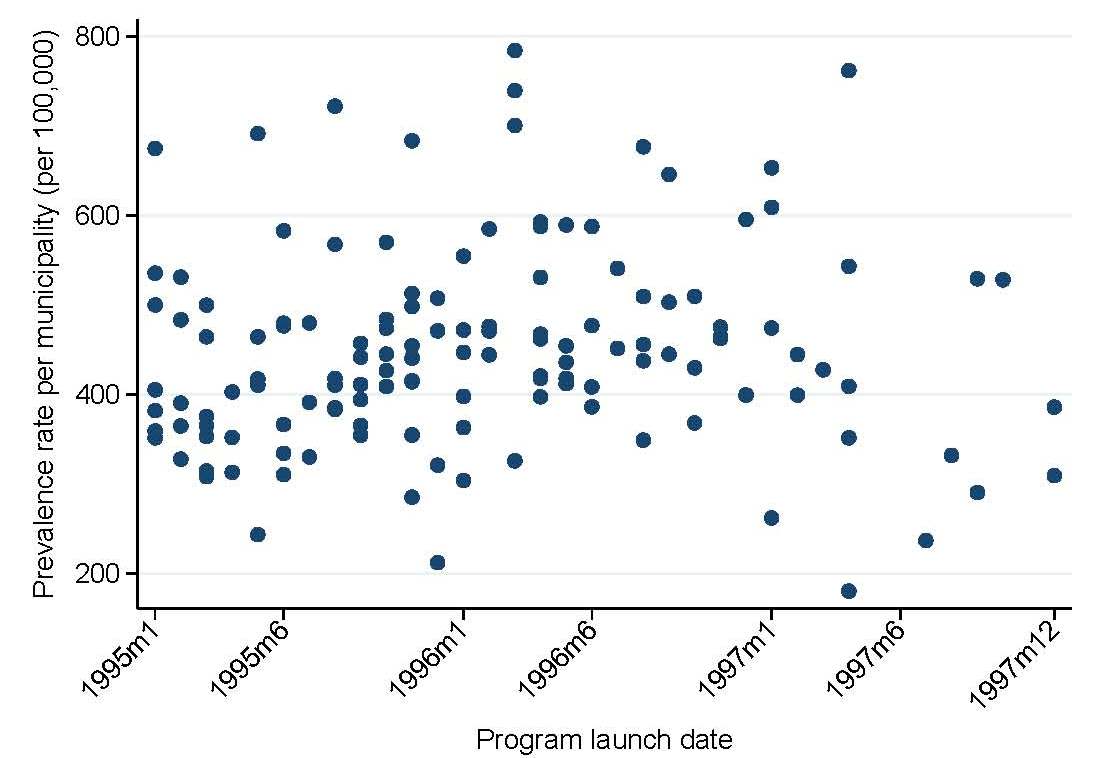


Program launch date is the month (m1 = January) when the program started to operate in a municipality. Prevalence rate is the municipality’s 5-year breast cancer prevalence as of January 1994.

Figure S5. Rate of screening take-up at first invitation against month of entry to the program


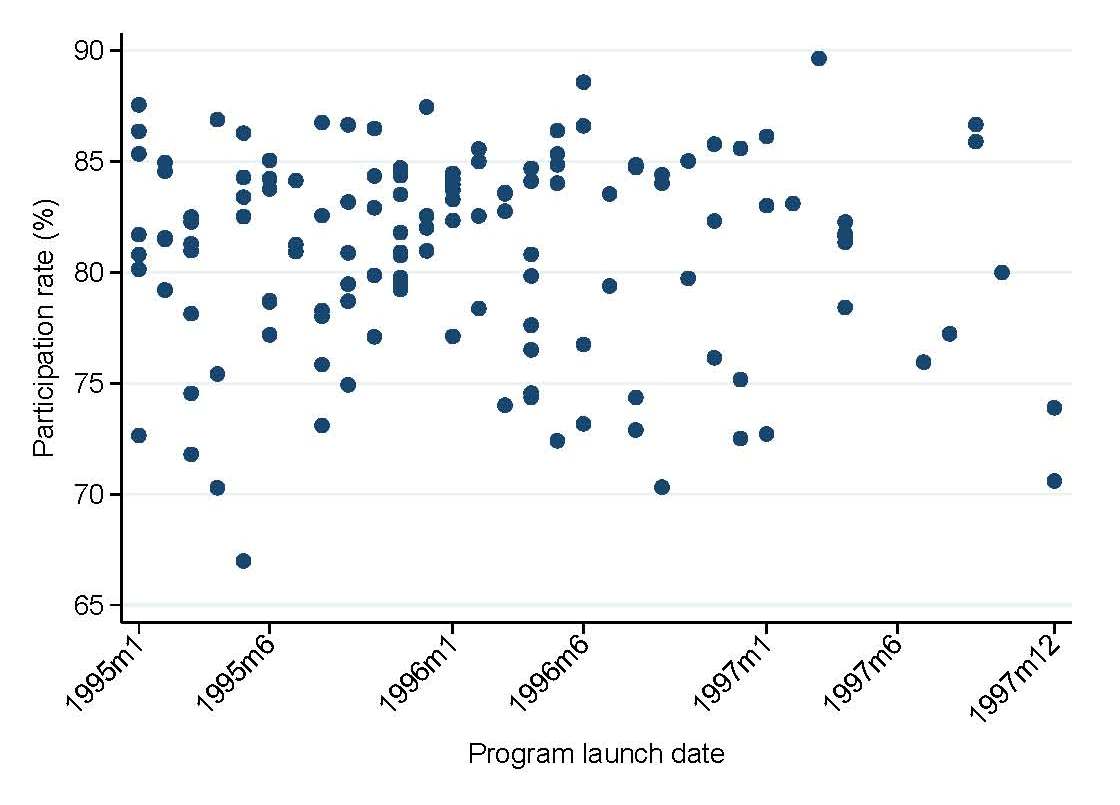


Program launch date is the month (m1 = January) when the program started to operate in a municipality. Participation rate is the percentage share of women in the eligible age range (50-69) that were screened when the program was launched in a municipality.

Table S1. Ordered probit models for a categorical dependent variable indicating the 6-month period (i.e. first half of 1995, …, second half of 1997) in which the municipality joined the program

|  | (I) | (II) | (III) | (IV) |
| --- | --- | --- | --- | --- |
| VARIABLES |  |  |  |  |
| Breast cancer incidence,1989-94 | -0.004  (0.004) |  |  | -0.004  (0.005) |
| Breast cancer prevalence, 1994 | 0.002  (0.001) |  |  | 0.002  (0.001) |
| Female population size, 1989-94 |  | 0.000  (0.007) |  | -0.000  (0.007) |
| Percentage of female population aged 50-69, 1989-94 |  | 0.041  (0.038) |  | 0.021  (0.043) |
|  |  |  |  |  |
| Percentage of women aged 50-69 participating in screening at first invitation |  |  | -0.008  (0.023) | -0.010  (0.026) |
| Observations | 138 | 138 | 133 | 133 |
| Pseudo R² | 0.004 | 0.003 | 0.000 | 0.004 |
| P-VALUES for JOINT HYPOTHESES |  |  |  |  |
| Incidence=Prevalence=0 | 0.439 |  |  | 0.532 |
| Population size=Percentage 50-69=0 |  | 0.564 |  | 0.872 |
| Incidence=Prevalence=Population size=Percentage 50-69=Percentage first=0 |  |  |  | 0.855s |

*Notes:* The dependent variable is a categorical variable indicating the 6-month period (i.e. first half of 1995, …, second half of 1997) in which the municipality joined the program. Ordered probit coefficients are reported. Breast cancer incidence is the municipality-specific median annual breast cancer incidence over the period 1989-1994. Breast cancer prevalence is the municipality-specific 5-year breast cancer prevalence as of January 1994. Female population size is expressed in 1000s and is the municipality average over the period 1989-1994. Percentage of female population aged 50-69 is the municipality average over the period 1989-94. Models III and IV are estimated on 133 observations because program participation information at first invitation is missing for 5 municipalities. P-Values are for tests of joint significance of the sets of covariates identified. Robust standard errors are in parentheses. * p < 0.1, ** p < 0.05, *** p < 0.01

Table S2. Models of the probability that a municipality entered the screening program in 1997 as opposed to 1995

|  | (Ia) | (Ib) | (IIa) | (IIb) | (IIIa) | (IIIb) | (IVa) | (IVb) |
| --- | --- | --- | --- | --- | --- | --- | --- | --- |
| VARIABLES | LPM | APE-logit | LPM | APE-logit | LPM | APE-logit | LPM | APE-logit |
| Breast cancer incidence,1989-94 | -0.005*  (0.003) | -0.005*  (0.003) |  |  |  |  | -0.005  (0.003) | -0.004  (0.003) |
| Breast cancer prevalence, 1994 | 0.001  (0.001) | 0.001  (0.001) |  |  |  |  | 0.001  (0.001) | 0.001  (0.001) |
| Female population size, 1989-94 |  |  | -0.000  (0.000) | -0.000  (0.000) |  |  | -0.000  (0.000) | -0.000  (0.000) |
| Percentage of female population aged 50-69, 1989-94 |  |  | 0.006  (0.022) | 0.005  (0.021) |  |  | 0.001  (0.021) | 0.004  (0.020) |
|  |  |  |  |  |  |  |  |  |
| Percentage of women aged 50-69 participating in screening at first invitation |  |  |  |  | -0.003  (0.011) | -0.003  (0.011) | -0.006  (0.011) | -0.006  (0.010) |
|  |  |  |  |  |  |  |  |  |
| Constant | 0.354*  (0.211) |  | 0.133  (0.402) |  | 0.453  (0.914) |  | 0.834  (1.061) |  |
| Observations | 86 | 86 | 86 | 86 | 83 | 83 | 83 | 83 |
| R² / Pseudo R² | 0.029 | 0.026 | 0.004 | 0.004 | 0.001 | 0.001 | 0.032 | 0.031 |
| P-VALUES for JOINT HYPOTHESES |  |  |  |  |  |  |  |  |
| Incidence=Prevalence=0 | 0.240 | 0.218 |  |  |  |  | 0.300 | 0.242 |
| Population size=Percentage 50-69=0 |  |  | 0.804 | 0.857 |  |  | 0.690 | 0.827 |
| Incidence=Prevalence=Population size=Percentage 50-69=Percentage first=0 |  |  |  |  |  |  | 0.721 | 0.684 |

*Notes:* The dependent variable is a binary indicator of organized screening starting in a municipality in 1997 (1), as opposed to 1995 (0). LPM: linear probability model coefficient. APE-logit: average partial effects from logistic regression. Breast cancer incidence is the municipality-specific median annual breast cancer incidence over the period 1989-1994. Breast cancer prevalence is the municipality-specific 5-year breast cancer prevalence as of January 1994. Female population size is expressed in 1000s and is the municipality average over the period 1989-1994. Percentage of female population aged 50-69 is the municipality average over the period 1989-94. Models III and IV are estimated on 83 observations because program participation information at first invitation is missing for 3 municipalities. P-Values are for tests of joint significance of the sets of covariates identified. Robust standard errors are in parentheses. * p < 0.1, ** p < 0.05, *** p < 0.01

Table S3. Effects on cumulative breast-cancer deaths per 100,000 women, 1995-2011

|  | (Ia) | (Ib) | (IIa) | (IIb) | (IIIa) | (IIIb) | (IVa) | (IVb) |
| --- | --- | --- | --- | --- | --- | --- | --- | --- |
| VARIABLES | LPM | APE-logit | LPM | APE-logit | LPM | APE-logit | LPM | APE-logit |
| Access to screening in 1997 as opposed to 1995 | 154***  (57) | 154***  (57) | 156***  (56) | 156***  (56) | 158***  (58) | 158***  (58) | 154***  (58) | 154***  (58) |
|  |  |  |  |  |  |  |  |  |
| Age on January 1995 (ref: 49) |  |  |  |  |  |  |  |  |
| 50 | 261  (173) | 261  (173) | 262  (173) | 262  (173) | 262  (172) | 262  (172) | 262  (172) | 262  (172) |
| 51 | 159  (173) | 159  (172) | 160  (173) | 160  (173) | 160  (172) | 160  (172) | 160  (172) | 160  (172) |
| 52 | 116  (149) | 116  (149) | 116  (150) | 116  (149) | 116  (149) | 116  (149) | 116  (150) | 115  (149) |
| 53 | 85  (134) | 85  (134) | 85  (134) | 85  (133) | 85  (134) | 85  (133) | 85  (133) | 85  (133) |
| 54 | 283*  (165) | 283*  (165) | 282*  (165) | 282*  (165) | 283*  (165) | 283*  (165) | 282*  (165) | 282*  (165) |
| 55 | 567***  (150) | 567***  (149) | 565***  (149) | 565***  (148) | 566***  (150) | 566***  (149) | 565***  (149) | 565***  (148) |
| 56 | 339**  (150) | 338**  (150) | 338**  (150) | 338**  (150) | 338**  (151) | 338**  (150) | 338**  (151) | 338**  (151) |
| 57 | 179  (152) | 179  (151) | 179  (151) | 179  (151) | 178  (152) | 178  (152) | 179  (152) | 179  (152) |
| 58 | 383**  (183) | 383**  (183) | 381**  (183) | 381**  (182) | 382**  (183) | 382**  (183) | 381**  (183) | 381**  (183) |
| 59 | 655***  (208) | 656***  (208) | 654***  (208) | 654***  (208) | 653***  (209) | 653***  (209) | 654***  (209) | 654***  (210) |
| 60 | 289**  (134) | 288**  (134) | 288**  (134) | 288**  (134) | 287**  (134) | 287**  (134) | 288**  (134) | 288**  (135) |
| 61 | 203  (155) | 202  (155) | 203  (155) | 203  (155) | 201  (156) | 201  (155) | 203  (156) | 203  (155) |
| 62 | 444***  (148) | 443***  (148) | 441***  (148) | 441***  (148) | 442***  (148) | 442***  (148) | 442***  (149) | 442***  (148) |
| 63 | 490***  (183) | 489***  (182) | 487***  (182) | 487***  (182) | 488***  (183) | 488***  (183) | 488***  (183) | 487***  (183) |
|  |  |  |  |  |  |  |  |  |
| Breast cancer incidence, 1989-94 |  |  | 4.95  (3.12) | 5.04  (3.18) |  |  | 4.98  (3.17) | 5.05  (3.22) |
| Breast cancer prevalence, 1994 |  |  | -0.44  (0.59) | -0.46  (058) |  |  | -0.43  (0.58) | -0.45  (0.58) |
| Female population size,1989-94 |  |  |  |  | -0.09  (1.04) | -0.08  (1.05) | -0.21  (0.95) | -0.16  (0.96) |
| Percentage of female population aged 50-69, 1989-94 |  |  |  |  | 8.93  (10.15) | 8.80  (9.98) | -0.20  (11.01) | -0.35  (10.94) |
| = |  |  |  |  |  |  |  |  |
| Constant | 1116***  (108) |  | 738***  (202) |  | 948***  (201) |  | 741***  (230) |  |
| Observations | 148,920 | 148,920 | 148,920 | 148,920 | 148,920 | 148,920 | 148,920 | 148,920 |
| P-VALUES ON JOINT HYPOTHESES |  |  |  |  |  |  |  |  |
| Incidence=Prevalence=0 |  |  | 0.104 | 0.102 |  |  |  |  |
| Population size=Percentage 50-69=0 |  |  |  |  | 0.675 | 0.673 |  |  |
| Incidence=Prevalence=Population size=Percentage 50-69=0 |  |  |  |  |  |  | 0.331 | 0.330 |

*Notes:* The dependent variable is a binary indicator for having died from breast cancer between 1995 and 2011. LPM: linear probability model. APE-logit: average partial effects from logistic regression. Coefficients (for LPM) and effects (for APE-logit) are rescaled to give estimated effects on number of breast-cancer deaths per 100,000 female inhabitants. First row gives effect of access to organized screening in 1997 as opposed to 1995. Breast cancer incidence is the municipality-specific median annual breast cancer incidence over the period 1989-1994. Breast cancer prevalence is the municipality-specific 5-year breast cancer prevalence as of January 1994. Female population size is expressed in 1000s and is the municipality average over the period 1989-94. Percentage of female population aged 50-69 is the average in the municipality over the period1989-94. P-Values are for tests of joint significance of the sets of covariates identified. Standard errors reported in parentheses are clustered over women who were living in the same municipality at the time of the first invitation. * p < 0.1, ** p < 0.05, *** p < 0.01

Table S4. Effect of the delayed access to screening program on cumulative breast-cancer deaths 1995-2011

|  | (Ia) | (Ib) |
| --- | --- | --- |
| VARIABLES | LPM | APE-logit |
| Period in which program started to operate in municipality |  |  |
| January-June 1995 (reference) |  |  |
| July-December 1995 | 35  (70) | 35  (70) |
| January-June 1996 | -44  (59) | -44  (59) |
| July-December 1996 | 10  (74) | 10  (74) |
| January-June 1997 | 163**  (72) | 163**  (72) |
| July-December 1997 | 186**  (77) | 186**  (77) |
| Age (reference: 49 in January 1995) |  |  |
| 50 in January 1995 | 141  (128) | 140  (128) |
| 51 in January 1995 | 93  (123) | 93  (123) |
| 52 in January 1995 | 196*  (118) | 196*  (118) |
| 53 in January 1995 | 166  (118) | 166  (118) |
| 54 in January 1995 | 236*  (125) | 236*  (125) |
| 55 in January 1995 | 327**  (114) | 327**  (114) |
| 56 in January 1995 | 266*  (136) | 266*  (136) |
| 57 in January 1995 | 128  (113) | 128  (113) |
| 58 in January 1995 | 324**  (136) | 324**  (135) |
| 59 in January 1995 | 503***  (143) | 502***  (143) |
| 60 in January 1995 | 302***  (105) | 302***  (105) |
| 61 in January 1995 | 332***  (125) | 332***  (125) |
| 62 in January 1995 | 425***  (116) | 425***  (116) |
| 63 in January 1995 | 450***  (134) | 450***  (134) |
|  |  |  |
| Constant | 1138***  (94) |  |
| Observations | 256,712 | 256,712 |

*Notes:* The dependent variable is a binary indicator for having died from breast cancer between 1995 and 2011. LPM: linear probability model. APE-logit: average partial effects from logistic regression. Coefficients (for LPM) and effects (for APE-logit) are rescaled to give estimated effects on number of breast-cancer deaths per 100,000 female inhabitants. Standard errors in parentheses are clustered over women who were living in the same municipality at the time of the first invitation. * p < 0.1, ** p < 0.05, *** p < 0.01

Table S5. Effect of access to screening program in 1997 as opposed to 1995 on cumulative breast-cancer deaths per 100,000 women, 1995-2011 – Robustness checks

|  | Control for province fixed effects^a^ | | Restrict to women not moving b/w municipalities^b^ | Placebo test on women aged 72-77 in Jan. 1995^c^ | Control for initial screening take-up rate^d^ | |
| --- | --- | --- | --- | --- | --- | --- |
| VARIABLES | No (I) | Yes (II) | (III) | (IV) | No (V) | Yes(VI) |
|  |  |  |  |  |  |  |
| LPM | 114  (76) | 139**  (57) | 126**  (57) | -101  (223) | 108*  (57) | 106*  (59) |
|  |  |  |  |  |  |  |
| APE-Logit | 114  (76) | 138**  (57) | 126**  (57) | -101  (222) | 108*  (57) | 105*  (59) |
| Included confounders | Age January 1995 | Age January 1995; Province fixed effects | Age January 1995 | Age January 1995 | Age January 1995 | Age January 1995; Participation rate |
| No. of municipalities | 36 | 36 | 86 | 86 | 83 | 83 |
| Observations | 69,254 | 69,254 | 143,424 | 33,081 | 143,356 | 143,356 |

*Notes:* The dependent variable is a binary indicator for having died from breast cancer between 1995 and 2011. Estimates shown for binary indicator of organized screening starting in a municipality in 1997, as opposed to 1995. LPM: linear probability model. APE-logit: average partial effects from logistic regression. Coefficients (for LPM) and effects (for APE-logit) are rescaled to give estimated effects on number of breast-cancer deaths per 100,000 female inhabitants. Age is controlled for by indicators of age in January 1995 in years, 49 (reference) and dummies for 50, 51 to 63. Standard errors are clustered over women who were living in the same municipality at the time of the first invitation, and reported in parentheses. * p < 0.1, ** p < 0.05, *** p < 0.01

^a^ The sample includes women living in 36 municipalities located in provinces where the program had not been fully implemented by January 1997

^b^ The sample includes women who did not move between municipalities during 1995-1997.

^c^ The sample includes women who were aged 72-77 in January 1995.

^d^ The sample includes women living in 83 municipalities for which we have data on the rate of screening take-up at the first invitation in a municipality.

Table S6. Effects on cumulative all-cause deaths per 100,000 women, 1995-2011

|  | (Ia) | (Ib) | (IIa) | (IIb) | (IIIa) | (IIIb) | (IVa) | (IVb) |
| --- | --- | --- | --- | --- | --- | --- | --- | --- |
| VARIABLES | LPM | APE-logit | LPM | APE-logit | LPM | APE-logit | LPM | APE-logit |
| Access to screening program in 1997 as opposed to 1995 | 10  (639) | 10  (638) | 6  (617) | 8  (616) | 331  (404) | 321  (411) | 392  (510) | 402  (521) |
|  |  |  |  |  |  |  |  |  |
| Age on January 1995 (ref: 49) |  |  |  |  |  |  |  |  |
| 50 | 412  (373) | 412  (373) | 414  (372) | 414  (373) | 414  (372) | 414  (372) | 431  (374) | 426  (375) |
| 51 | 748**  (357) | 748**  (357) | 749**  (357) | 749**  (357) | 777**  (365) | 768**  (360) | 782**  (366) | 772**  (360) |
| 52 | 865***  (303) | 865***  (302) | 862***  (303) | 863***  (303) | 878***  (302) | 873***  (304) | 878***  (301) | 875***  (304) |
| 53 | 2,067***  (381) | 2,067***  (381) | 2,069***  (382) | 2,069***  (382) | 2,087***  (383) | 2,083***  (378) | 2,082***  (382) | 2,080***  (376) |
| 54 | 3,129***  (372) | 3,129***  (372) | 3,127***  (371) | 3,128***  (372) | 3,133***  (370) | 3,133***  (361) | 3,123***  (371) | 3,126***  (358) |
| 55 | 4,383***  (436) | 4,383***  (436) | 4,376***  (435) | 4,377***  (434) | 4,413***  (431) | 4,411***  (428) | 4,396***  (436) | 4,398***  (428) |
| 56 | 4,817***  (467) | 4,817***  (465) | 4,814***  (467) | 4,815***  (469) | 4,810***  (470) | 4,812***  (488) | 4,796***  (467) | 4,801***  (488) |
| 57 | 7,126***  (439) | 7,125***  (438) | 7,127***  (438) | 7,128***  (438) | 7,129***  (446) | 7,130***  (483) | 7,108***  (443) | 7,117***  (487) |
| 58 | 8,115***  (471) | 8,115***  (470) | 8,107***  (471) | 8,107***  (468) | 8,102***  (463) | 8,101***  (421) | 8,082***  (464) | 8,084***  (424) |
| 59 | 10,436***  (495) | 10,436***  (495) | 10,432***  (495) | 10,433***  (499) | 10,433***  (494) | 10,433***  (501) | 10,406***  (487) | 10,407***  (493) |
| 60 | 11,765***  (441) | 11,765***  (441) | 11,761***  (441) | 11,762***  (443) | 11,758***  (437) | 11,758***  (393) | 11,726***  (432) | 11,724***  (392) |
| 61 | 13,303***  (518) | 13,302***  (518) | 13,307***  (517) | 13,310***  (521) | 13,281***  (504) | 13,275***  (478) | 13,250***  (501) | 13,240***  (468) |
| 62 | 16,401***  (425) | 16,401***  (425) | 16,393***  (424) | 16,391***  (423) | 16,359***  (419) | 16,340***  (474) | 16,336***  (419) | 16,316***  (466) |
| 63 | 18,916***  (660) | 18,916***  (659) | 18,908***  (662) | 18,905***  (667) | 18,864***  (662) | 18,834***  (734) | 18,848***  (661) | 18,821***  (751) |
|  |  |  |  |  |  |  |  |  |
| Breast cancer incidence, 1989-94 |  |  | 25.04  (17.93) | 25.16  (18.00) | 26.02  (16.64) | 26.87  (17.09) |  |  |
| Breast cancer prevalence, 1994 |  |  | -3.74  (4.67) | -3.75  (4.67) | -3.50  (3.18) | -3.56  (3.27) |  |  |
| Female population size, 1989-94 |  |  |  |  | 34.91***  (6.52) | 33.49***  (6.17) | 32.36***  (7.43) | 30.70***  (7.02) |
| Percentage of female population aged 50-69, 1989-94 |  |  |  |  | -171.08  (108.48) | -173.45  (108.30) |  |  |
|  |  |  |  |  |  |  |  |  |
| Constant | 8,592***  (529) |  | 7,338***  (1,174) |  | 9,457***  (1,720) |  | 7,717***  (349) |  |
| Observations | 148,920 | 148,920 | 148,920 | 148,920 | 148,920 | 148,920 | 148,920 | 148,920 |

*Notes:* The dependent variable is a binary indicator for having died from any cause between 1995 and 2011. LPM: linear probability model estimates. APE-logit: average partial effects from logistic regression. Coefficient (for LPM) and effects (for APE-logit) are rescaled to give estimated effects on number of deaths from any cause per 100,000 female inhabitants. First three rows give effects of delay to access organized screening in 1997 as opposed to 1995. Breast cancer incidence is the municipality-specific median annual breast cancer incidence over the period 1989-1994. Breast cancer prevalence is the municipality-specific 5-year breast cancer prevalence as of January 1994. Female population size is expressed in 1000s and is the municipality average over the period 1989-94. Percentage of female population aged 50-69 is the average in the municipality over the period 1989-94. Standard errors reported in parentheses are clustered over women who were living in the same municipality at the time of the first invitation. * p < 0.1, ** p < 0.05, *** p < 0.01

**REFERENCES**

1. Otto SJ, Fracheboud J, Looman CW, et al. Initiation of Population-Based Mammography Screening in Dutch Municipalities and Effect on Breast Cancer Mortality: A Systematic Review. Lancet 2003; 361: 1411-7.
2. Dutch Cancer Registry managed by the Netherlands Comprehensive Cancer Organisation
3. Zelen M. Relations between incidence, prevalence and time with disease, Part I. 2005, http://www.pitt.edu/~super1/lecture/lec19481/index.htm.
4. Belloni A, Chernozhukov V. Hansen C. Inference on Treatment Effects after Selection among High-Dimensional Controls. Rev Econ Stud 2014, 81: 608-50.
5. van Leeuwen FE, van den Belt-Dusebout A, Benraadt J et al. Risk of Endometrial Cancer After Tamoxifen Treatment of Breast Cancer. Lancet 1994; 343: 448-52.
6. Bergman L, Beelen ML, Gallee et al. Risk and Prognosis of Endometrial Cancer after Tamoxifen for Breast Cancer. Lancet 2000; 356: 881-7.
7. Early Breast Cancer Trialists' Collaborative Group. Favourable and Unfavourable Effects on Long-Term Survival of Radiotherapy for Early Breast Cancer: An Overview of the Randomized Trials. Lancet 2000; 355: 1757-70.
8. Harris EE, Correa C, Hwang WT et al. Late Cardiac Mortality and Morbidity in Early-Stage Breast Cancer Patients after Breast-Conservation Treatment. J Clin Oncol 2006; 24: 4100-6.
9. Welch HG, Prorok PC, O’Malley J, Kramer B. Breast-Cancer Tumor Size, Overdiagnosis, and Mammography Screening Effectiveness. NEJM 2016; 3715: 1438-1447.
10. Honore BE, Lleras-Muney A. Bounds in Competing Risks Models and the War on Cancer. Econometrica 2006; 74: 1675-98.
11. Chapman JAW, Meng D, Shepherd L et al. Competing Causes of Death From a Randomized Trial of Extended Adjuvant Endocrine Therapy for Breast Cancer. J Natl Cancer Inst 2008; 100: 252-60.
12. Penston J. Should We Use Total Mortality Rather than Cancer Specific Mortality to Judge Cancer Screening Programmes? Yes. BMJ 2011; 343: d6395.
13. Black WC, Haggstrom DA, Welch HG. All-Cause Mortality in Randomized Trials of Cancer Screening. J Natl Cancer Inst 2002; 94: 167-73.
14. Boniol M, Autier P, Gandini S. Melanoma mortality following skin cancer screening in Germany. BMJ Open 2015; 5:e008158.
15. Stang A, Jöckel K-H. Does skin cancer screening save lives? A detailed analysis of mortality time trends in Schleswig-Holstein and Germany. Cancer 2016; 122: 432-437.
16. Harteloh P, de Bruin K, Kardaun J. The Reliability of Cause-of-Death Coding in the Netherlands. Eur J Epidemiol 2010; 25: 531-8.
17. Van Schoor G, Moss S, Otten J, et al. Increasingly Strong Reduction in Breast Cancer Mortality Due to Screening. Brit J Cancer 2011; 104: 910-4.
18. Otto SJ, Fracheboud J, Verbeek AL, et al. Mammography Screening and Risk of Breast Cancer Death: A Population-Based Case-Control Study. Cancer Epidemiol Biomarker Prev 2012, 21: 66-73.
